# Interaction between Different Implant Surfaces and Liquid Fibrinogen: A Pilot In Vitro Experiment

**DOI:** 10.1155/2021/9996071

**Published:** 2021-07-05

**Authors:** Catherine X. Andrade, Marc Quirynen, David R. Rosenberg, Nelson R. Pinto

**Affiliations:** ^1^Department of Periodontology and Implantology, Faculty of Dentistry, Universidad de Los Andes, Santiago, Chile; ^2^Section of Periodontology, Department of Oral Health Science, University Hospitals, KU Leuven, Belgium; ^3^Department of Oral and Maxillofacial Surgery, Faculty of Dentistry, Universidad de Los Andes, Santiago, Chile

## Abstract

**Background:**

Platelet concentrates like leucocyte- and platelet-rich fibrin (L-PRF) have been widely evaluated in different oral surgical procedures to promote the healing process. However, liquid L-PRF products such as liquid fibrinogen have been poorly explored, especially in the biomimetic functionalization of dental implants. The aim of this in vitro study is to evaluate the interaction between 5 different dental implant surfaces and liquid fibrinogen.

**Methods:**

Five commercially available dental implants with different surfaces (Osseospeed™, TiUnite™, SLActive®, Ossean®, and Plenum®) were immersed for 60 minutes in liquid fibrinogen obtained from healthy donors. After this period, the implants were removed and fixed for scanning electron microscopy (SEM).

**Results:**

All dental implants were covered by a fibrin mesh. However, noticeable noncontact areas were observed for the Osseospeed™, TiUnite™, and SLActive® surfaces. On the other hand, Ossean® and Plenum® surfaces showed a dense and uniform layer of fibrin covering almost the entire implant surface. The Osseospeed™, TiUnite™, and SLActive® surfaces presented with lower blood cell numbers inside the fibrin mesh compared with the others. Moreover, at higher magnification, thicker fibrin fibers were observed in contact with Ossean® and Plenum® surfaces. The Plenum ®surface showed the thickest fibers which also inserted and interconnect to the microroughness.

**Conclusion:**

The initial contact between an implant surface and the fibrin network differs significantly among different implant brands. Further studies are necessary to explore the clinical impact of these observations in the osseointegration process of dental implants.

## 1. Introduction

Dental implants are considered a safe treatment option for partially or fully edentulous patients with high long-term survival rates [1]. However, osseointegration is less predictable in some conditions such as poorly controlled diabetes, tobacco consumption, bisphosphonate medication, and radiotherapy [2, 3].

A fundamental and early step in peri-implant hard tissue healing is the formation of a stable fibrin clot allowing the migration of different cells toward the bone-implant interface [4].

In order to improve osseointegration, physiochemical characteristics of implant surfaces such as topography, wettability, and surface energy have been widely studied and modified [5–7]. Different biomimetic approaches for the functionalization of implant surfaces have also been explored using agents like calcium phosphate, hydroxyapatite (HA), bone morphogenetic proteins (BMPs), and growth factors in order to promote osteoinduction, osteoconduction, and osteogenesis [8–10].

These considerations could be especially relevant in patients who have shown higher marginal bone loss rates around implants and lower survival rates such as patients previously treated for periodontitis [11, 12].

Platelet concentrates like leucocyte- and platelet-rich fibrin (L-PRF) and flowable products such as liquid fibrinogen are rich in fibronectin and vitronectin, two proteins with important roles in the platelet function (adhesion, aggregation, and activation) and later cell adhesion to the extracellular matrix in the healing process [13, 14]. Additionally, these platelet concentrates are able to release growth factors (GF) such as transforming growth factor-*β*1 (TGF-*β*1), vascular endothelial growth factor (VEGF), and platelet-derived growth factor-AB (PDGF-AB) promoting angiogenesis and cell migration and differentiation [15, 16]. Thus, platelet concentrates could be an effective agent to get a biomimetic autologous functionalization of implant surfaces optimizing the osseointegration process.

Öncü and Alaaddinoglu observed statistically higher “ImplantStability Quotient” (ISQ) values in implants coated with L-PRF exudate and applying an L-PRF membrane in the implant osteotomies compared those without L-PRF at week 1 and week 4 after implant placement without an intermediate drop in ISQ for L-PRF sites [17]. These observations were confirmed in another randomized clinical trial (RCT) where a slight increase in ISQ was observed for the osteotomies filled with L-PRF at 1 week compared to a drop of 2 ISQ units in the osteotomies without L-PRF. Additionally, higher increases were observed after 1 month for the L-PRF group [18].

Superior bone density was also reported in osteotomies treated with L-PRF before implant placement compared to sites without L-PRF after 6 and 12 weeks [19].

Currently, the evidence about the use of platelet concentrates in this application is limited, requiring further studies. The aim of this in vitro study is to evaluate the interaction between 5 different dental implants surfaces and liquid fibrinogen, as a first step in the formation of a fibrin network.

## 2. Materials and Methods

### 2.1. Liquid Fibrinogen Preparation

Four venous blood samples from healthy donors were collected in 9 ml noncoated vacutainer tubes without anticoagulants (white cap). The samples were centrifuged in a table centrifuge (IntraSpin™, Intra-lock®, Florida, USA) at 2700 rpm for 3 minutes, according to the protocol used by Andrade et al. [20]. The yellow liquid at the top of the white cap tubes (liquid fibrinogen) was aspirated with a sterile syringe, (avoiding red blood cells) and immediately transferred into 5 Eppendorf tubes (1.5 ml).

### 2.2. Dental Implant Processing

Five commercially available dental implants with different surfaces obtained by specific manufacturing techniques were analyzed: Osseospeed™ (AstraTech, Mölndal, Sweden), TiUnite™ (Nobel Biocare, Gothenburg, Sweden), SLActive® (ITI Straumann, Basel, Switzerland), Ossean® (Intra-Lock, Boca Raton, FL, USA), and Plenum® (PlenumBioengenharia, Sao Paulo, Brazil).  Table 1 shows the surface treatment of each dental implant evaluated according to the description reported by Dohan et al. (2011). For the Plenum® surface, the information was provided by the manufacturing company.

**Figure 1:**
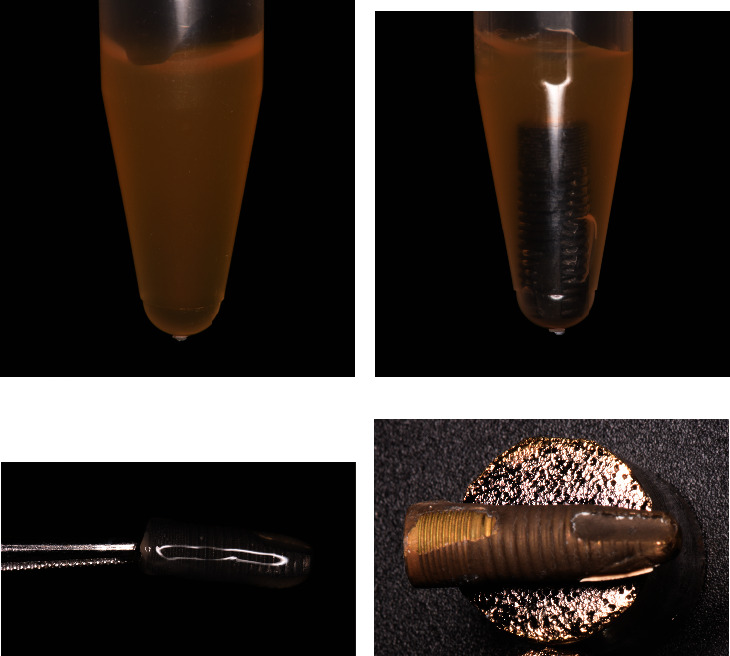
(a) Liquid fibrinogen transferred in the Eppendorf tube. (b) Dental implant immersed in liquid fibrinogen for 60 min. (c) Dental implant carefully removed together with adhering fibrin clot to be fixed in 2% glutaraldehyde in 0.1 M sodium cacodylate buffer. (d) Sample prepared for the SEM analysis.

The entire implant was immersed in liquid fibrinogen for 60 minutes at room temperature. After this period, the implants were carefully removed preserving the fibrin clot attached to them. The fibrin clot was not manipulated in any way. The samples were fixed immediately in 2% glutaraldehyde in 0.1 M sodium cacodylate buffer (pH 7.4). Then, the specimens were prepared for an analysis with a JSM 6380LV–JEOL scanning electron microscope (SEM) at different magnifications (×500, ×1000, ×2000, and ×5000) and an acceleration voltage of 20 kV (Figure 1).

## 3. Results

### 3.1. Macroscopic Observations

The formation of bubbles when immersing the implants in liquid fibrinogen was observed on the Osseospeed™, TiUnite™, and SLActive® surfaces. After 60 minutes, these bubbles were associated with areas without fibrin coverage.

### 3.2. SEM Image Interpretation

**Figure 2:**
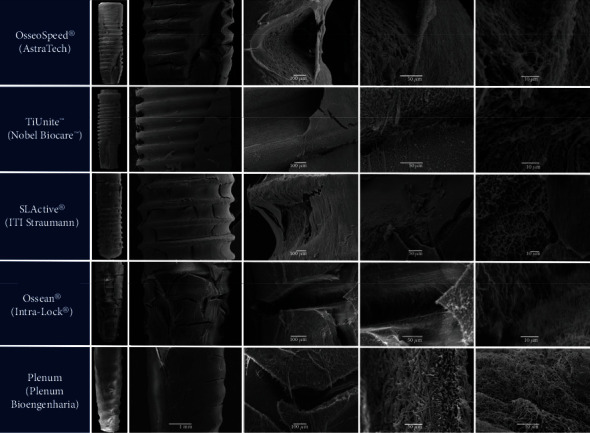
Implant surfaces analyzed by SEM at different magnifications.

**Figure 3:**
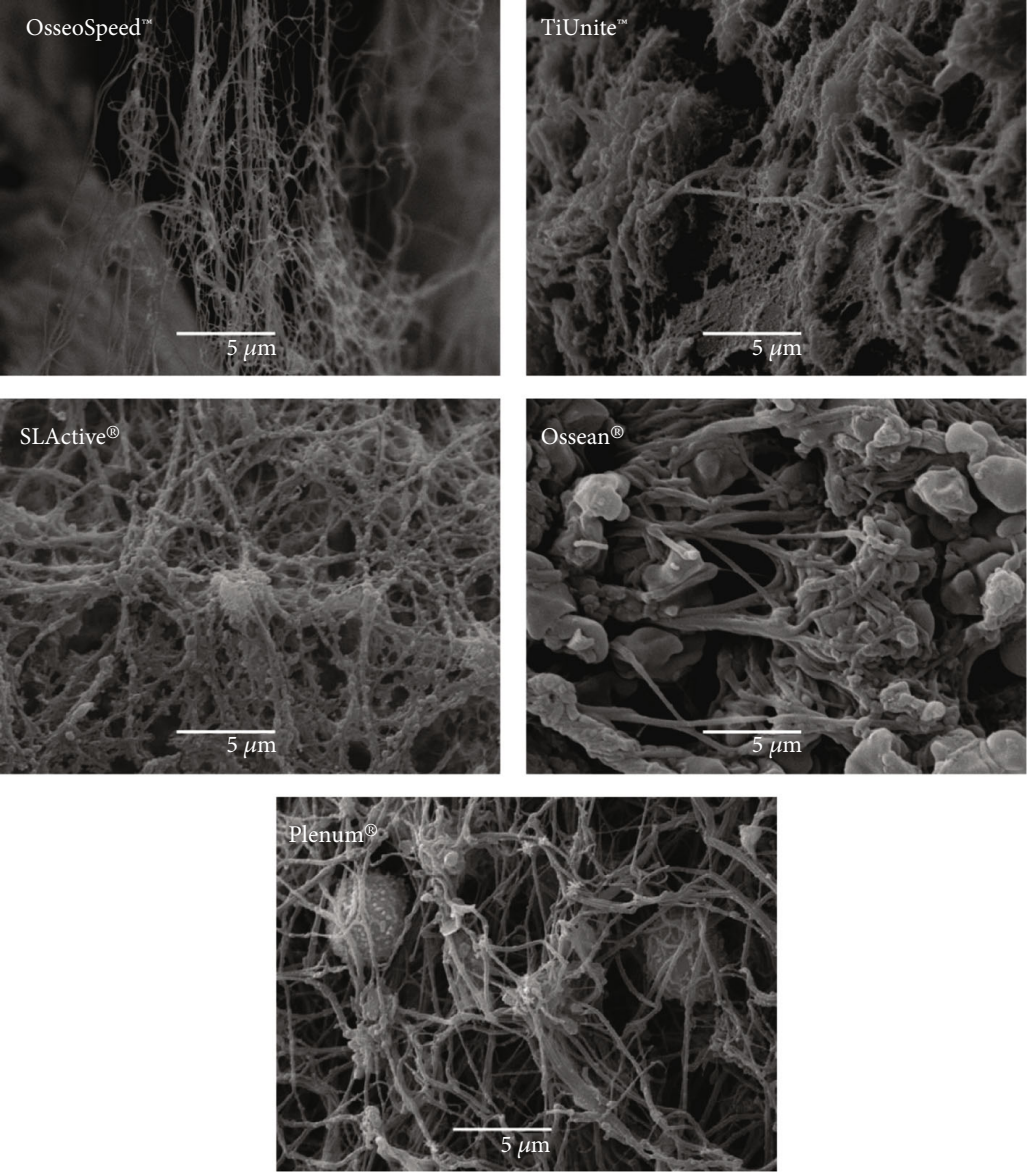
At higher magnification, fewer cells were observed on the Osseospeed™, TiUnite™, and SLActive® surfaces compared to the Ossean® and Plenum®. Especially, Plenum® surface showed several leucocytes trapped a dense fibrin mesh close to the implant surface.

Despite the fact that all the implants were covered with a fibrin mesh, clear differences were observed both at low and high magnification. Osseospeed™, TiUnite™, and SLActive® surfaces showed significant zones without fibrin coverage in contrast to the Ossean® and Plenum® surfaces, where a dense and uniform layer of fibrin covered almost the entire implant surface (Figure 2). Additionally, Osseospeed™, TiUnite™, and SLActive® surfaces showed a thinner fibrin layer with less fibrin fibers running towards the implant surface, when compared to the Ossean® and Plenum® surfaces. The thickness of the fibers and density of the fibrin mesh were clearly higher in the Ossean® and Plenum® surfaces. Furthermore, these surfaces showed more blood cells trapped inside of the fibrin mesh (Figure 3).

**Figure 4:**
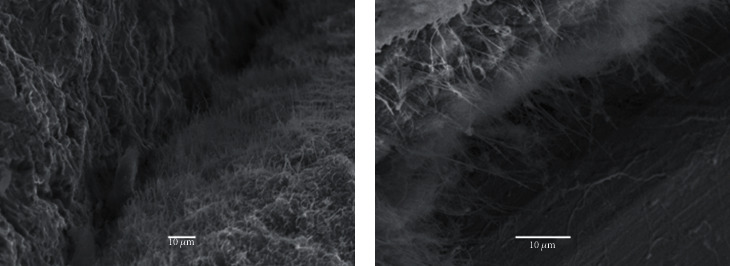
(a) Fibers running parallel to the implant surface. (b) Fibers running perpendicularly.

The fibrin fibers on Osseospeed™, TiUnite™, and SLActive® surfaces ran mostly parallel to the implant surface in contrast to the Ossean® and Plenum® surfaces where part of fibers ran perpendicular to the surface (Figure 4).

**Figure 5:**
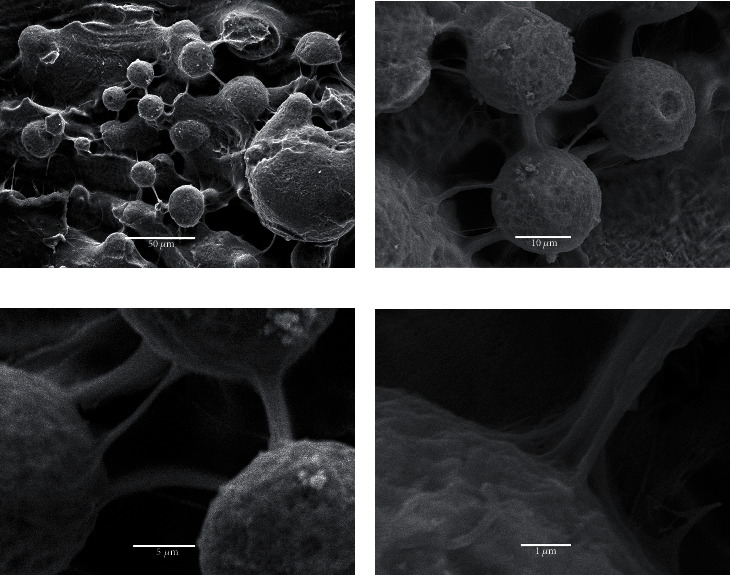
Interaction between fibrin and Plenum surfaces at different magnifications. Thick fibers inserted into the microroughness and/or making connections between the microroughness on the surface.

On the Plenum® surface, large number of fibers seemed to be inserted and interconnected with the microroughness on the surface. This was never observed in any of the other implants. These fibers also had a larger diameter (Figure 5).

## 4. Discussion

This in vitro study evaluated the interaction of different implant surfaces with a liquid L-PRF product (liquid fibrinogen) in order to elucidate if the implant surface characteristics (topography, wettability, coating, etc.) had an impact on the fibrin mesh characteristics obtained from this autologous blood product, so that it could be eventually used as an effective biomimetic functionalization of dental implants.

All implant surfaces in this study formed a stable fibrin mesh when in contact with liquid fibrinogen. However, macroscopic and microscopic differences were observed.

Osseospeed™, TiUnite™, and SLActive® surfaces showed some bubble formation when immersed in liquid fibrinogen, which was subsequently expressed in areas without fibrin. This reaction might be associated with a lower hydrophilicity compared with the other surfaces. On the other hand, Ossean® and Plenum® surfaces showed a better coverage with a thick and dense fibrin layer with more cells trapped inside.

The micro/nanotopography of implant surfaces was found to be a key aspect for these results. Surfaces with more texture at the nanoscale increase their surface energy, and this also increases the wettability to blood and as such the diffusion and attachment of fibrin and matrix proteins. The nanopatterning could also modulate the cell behavior, stimulating cell proliferation and differentiation [7].

The Osseospeed™ surface is considered heterogenous with a moderate microroughness covered by a nanoroughness but impacted by large TiO2 residual blasting particles with a very smooth surface. TiUnite™ is a microporous surface, smooth on the nanoscale, and with extended cracks associated with the anodization process, and the SLActive® surface has a moderate microroughness and a significant nanotexturization. However, the morphology of this coating is very heterogeneous [21].

On the other hand, the Ossean® surface obtained by blasting/etching and with unknown postprocessing, presents a thicker layer of TiO2 and low levels of calcium phosphate homogeneous over all surfaces. It exhibits a minimal microroughness close to the moderate level and is covered completely with a nanoroughness. This surface is homogeneous in chemistry and topography and is considered as a fractal surface [21].

Plenum® is a new implant obtained by 3D printing using an additive process applying successive layers of titanium powder that are fused by a laser beam. The final surface has unique characteristics that mimic the microstructure of a trabecular bone, stimulating cell adhesion and new bone formation [22]. This special topography resulted in the presence of thicker fibrin fibers inserted and interconnected between its microroughness.

A previous clinical study showed in an animal model with immediate implant placement a significantly higher bone area fraction occupancy (BAFO) on the Ossean® surface wrapped with L-PRF compared with a dual acid-etched (DAE) surface [23]. The superior result for the Ossean® surface was explained by its micrometer/nanometer-scale textured topography which seemed to improve the interaction with the L-PRF mesh and at the same time stimulate the expression of osteogenic genes [23, 24].

Similar results were reported by Lollobrigida et al. (2018). They evaluated the fibrin formation on titanium discs with rough fractal nanosurfaces (Ossean®) and machined surfaces immersed in liquid fibrinogen and L-PRF exudate. They observed a denser fibrin network and more blood cells when using liquid fibrinogen instead of L-PRF exudate, but more specifically an increased retention of fibrin in micro/nanorough samples compared to machined surfaces, resulting in a thicker coating [25].

Knowing the importance of the establishment of a fibrin clot to promote cell migration and differentiation during the osseointegration process, the platelet concentrates could contribute all necessary elements such as fibrin mesh, platelets, leucocytes and growth factors, and also important proteins like fibronectin and vitronectin to promote these events [26].

Therefore, liquid fibrinogen could be a feasible and efficient method to obtain an autologous biomimetic functionalization of implant surfaces with a simple protocol while considering the implant surface characteristics in order to optimize the results.

This application could be especially favorable in patients with bone healing alterations such as smokers, those under radiotherapy, patients taking bisphosphonate medication, or immediate implant placement where a gap exists between the alveolar bone and the implant surface.

A limitation of this study could be the observational nature of data and the limited implant surfaces evaluated. Other implants with special nanosurfaces such laser microtexture surface (LMS) with successful clinical results maintaining the alveolar bone, obtaining a high quality of tissue sealing, and preventing peri-implantitis, should be considered in future research [27–29] .

However, the present study is the first to evaluate the interaction of a liquid L-PRF product (liquid fibrinogen) over dental implants, showing a most realistic scenario.

Further studies are necessary to evaluate other factors that could influence biomimetic functionalization with liquid L-PRF products and explore the clinical impact of these observations in the osseointegration process and success rate of dental implants placed with this technique.

## 5. Conclusion

This preliminary in vitro study showed how implant surface characteristics like topography, wettability, and coatings might influence the interaction between the implant surface and the fibrin mesh obtained from liquid fibrinogen, suggesting that some implant surfaces are more suitable for a biomimetic functionalization with platelet concentrates. Considering the limitation of the present study: the observational design, not all implant brands or surfaces analyzed, and not all type of donor patients, further studies are necessary to expand this analysis and to explore the clinical impact of these observations.

## Figures and Tables

**Table 1:** Surface treatment of implants evaluated.

Implant surface (comercial brand)	Surface treatment classification	Microtopography	Nanotopography	Global architecture
OsseoSpeed™ (Astra Tech)	Combined Physical: Sandblasting (titanium oxide) Chemical: fluoride modified	Rough Moderate Flattened out	Rough	Nonfractal Heterogeneous TiO_2_–random particle

TiUnite™ (Nobel Biocare™)	Chemical: anodization	Porous Moderate Extra rugged	Smooth	Nonfractal Homogeneous Extended cracks

SLActive® (ITI Straumann)	Combined Physical: Sandblasted Chemical: acid etched	Rough Moderate Rugged	Sodium chloride particle	Nonfractal Heterogeneous

Ossean® (Intra-Lock®)	Combined Physical: Sandblasting (titanium oxide) Chemical: acid etched, calcium phosphate (molecular impregnation)	Rough Minimal Flattened out	Rough	Fractal Homogeneous

Plenum® (PlenumBioengenharia)	Biomimetic surface Additive manufactured (laser microfusion of Ti particles grade 23)	Rough Moderate Extra rugged	Rough	Fractal Homogeneous

## Data Availability

All data analyzed during this study are available from the corresponding author on reasonable request.
